# Shampooing Versus Massage

**Published:** 1889-03-23

**Authors:** Hendley Jeypoor

**Affiliations:** India


### Shampooing versus Massage.

By Mrs. Hendley, Jeypoor, India.

Very much is talked, heard, and written concerning the so-called new treatment denominated massage. Having submitted ourselves to the operation, the suspicion that it was merely the introduction into the West of the process so long familiar to dwellers in the East as shampooing, was confirmed. Massage from personal experience seems only a second-rate shampooing, and a poor imitation of it. Shampooing is an art daily, almost hourly, exercised in the homes of Asiatics. It is an art in which almost every Hindu woman is an adept, both by daily practice and inheritance. In whatever else the anomalous individual whom we distinguish by the name of Ayah may be found deficient, almost to a certainty she is found both thoroughly competent and always ready and willing to exercise her gift of shampooing on behalf of those whom she serves. A good Ayah, while crooning over her restless charge, charms it to slumber by combining monotonous chant with a continous gentle pressure of the weary little limbs, and soon the profound sleep which ensues demonstrates the efficacy of the means. The baby's departure to the land of dreams is the signal for its guardian to roll herself up and follow suit. When languor and the aching and weariness of fever and an enervating climate are doing their deadly work on the sensitive frames of European females, then the Ayah's art proves invaluable. How many weary, sleepless nights have been averted by her persistent, patient efforts to restore tone and energy to the muscles, circulation to the blood, and firmness to the bones? Beginning with the spine, she slowly presses between her firm hands each part in turn. Then when your back no longer feels only a succession of disjointed and aching muscles, she places you on your face or back and presses together your aching sides, till each rib seems once more to take to its natural function of keeping internal organs in place. Then commencing from the finger tips, she slowly carries up to the shoulder-blade the restored sense of being, and then the shampooing is recommenced with each toe, and carried up to the thigh. As the process goes on a delicious sense of dreamy repose steals over the patient, and often, as in the case of the weary "baby Sahib," the Ayah is rewarded by finding her "mem Sahib" has passed beyond the sphere of her ministrations into the blissful land of dreams.

Massage, we are told, requires a long and expensive training in those that engage in it. It is consequently a very costly luxury for those who benefit by it. It is to be feared, therefore, that whatever its advantages and blessings, these are likely to be available only for the wealthy. We all know too well that the great majority of sufferers from those long and tedious illnesses where massage would be so much desiderated, are of a class to whom it would be all but unattainable. In these days of financial difficulty, hospital committees would hesitate to sanction the expenditure that would be necessary to attach to each of them a staff of experienced "professors" of massage; but an experience of many years in the East convinces us that an incalculable amount of suffering, especially that form of it peculiar to tedious cases and long illnesses—restlessness and sleeplessness—might be minimised, if not avoided, by the free practice of shampooing in our hospitals. It is to this end that we venture to make the following suggestion, which doubtless might be improved upon by further experience. It may not be known to the general public that the great steamers traversing the so-called overland route from India bring annually to British shores a host of those experts in the art of shampooing—Indian Ayahs! These women, as a rule, pass the summer months in England, in hopes of a return engagement with families taking out young children to India in the autumn. It is often extremely difficult to provide suitable homes for these strangers, so kind and so dear to every Anglo-Indian child. Could not the experiment be made of securing the services of these most useful, obliging, and non-exigent persons for the great metropolitan general hospitals. The offer of shelter and food, and a very small monthly retaining fee, would, we think, be regarded as a boon by these homeless strangers, and we venture to say their gentle, kindly ways, and their simple but beneficent gift, would cheer and soothe weary hours of suffering to many poor sleepless patients. Naturally, the long summer months are those in which the sick suffer most from restlessness and languor. To bring to the bedside of such the kindly though swarthy face of an Indian Ayah, and bid her give him rest and slumber, would be, to say the least, a harmless and very inexpensive experiment, and might fulfil two good ends, viz., those, (1) Of providing a safe home with some remuneration to the stranger within our gates, so that their sojourn may not be barren of interest and profit to them; (2) and chiefly by bringing within the reach of the poorest the benefits in part of the treatment called massage.

